# Effects of Pregabalin on Central Sensitization in Patients with Chronic Pancreatitis in a Randomized, Controlled Trial

**DOI:** 10.1371/journal.pone.0042096

**Published:** 2012-08-06

**Authors:** Stefan A. W. Bouwense, Søren S. Olesen, Asbjørn M. Drewes, Jan-Werner Poley, Harry van Goor, Oliver H. G. Wilder-Smith

**Affiliations:** 1 Pain and Nociception Neuroscience Research Group, Department of Surgery, Radboud University Nijmegen Medical Center, Nijmegen, The Netherlands; 2 Mech-Sense, Department of Gastroenterology and Hepatology, Aalborg Hospital, Aarhus University Hospital, Aalborg, Denmark; 3 Center for Sensory-Motor Interaction (SMI), Department of Health Science and Technology, Aalborg University, Aalborg, Denmark; 4 Department of Gastroenterology and Hepatology, Erasmus MC, University Medical Center, Rotterdam, The Netherlands; 5 Pain and Nociception Neuroscience Research Group, Department of Anaesthesiology, Pain and Palliative Care, Radboud University Nijmegen Medical Center, Nijmegen, The Netherlands; The James Cook University Hospital, United Kingdom

## Abstract

**Background:**

Intense abdominal pain is the dominant feature of chronic pancreatitis. During the disease changes in central pain processing, e.g. central sensitization manifest as spreading hyperalgesia, can result from ongoing nociceptive input. The aim of the present study is to evaluate the effect of pregabalin on pain processing in chronic pancreatitis as assessed by quantitative sensory testing (QST).

**Methods:**

This randomized, double-blind, placebo-controlled trial evaluated effects of pregabalin on pain processing. QST was used to quantify pain processing by measuring thresholds to painful electrical and pressure stimulation in six body dermatomes. Descending endogenous pain modulation was quantified using the conditioned pain modulation (CPM) paradigm to elicit a DNIC (diffuse noxious inhibitory controls) response. The main effect parameter was the change in the sum of all body pain threshold values after three weeks of study treatment versus baseline values between both treatment groups.

**Results:**

64 patients were analyzed. No differences in change in sum of pain thresholds were present for pregabalin vs. placebo after three weeks of treatment. For individual dermatomes, change vs. baseline pain thresholds was significantly greater in pregabalin vs. placebo patients for electric pain detection threshold in C5 (P = 0.005), electric pain tolerance threshold in C5 (P = 0.04) and L1 (P = 0.05), and pressure pain tolerance threshold in T4 (P = 0.004). No differences were observed between pregabalin and placebo regarding conditioned pain modulation.

**Conclusion:**

Our study provides first evidence that pregabalin has moderate inhibitory effects on central sensitization manifest as spreading hyperalgesia in chronic pancreatitis patients. These findings suggest that QST can be of clinical use for monitoring pain treatments in the context of chronic pain.

**Trial Registration:**

ClinicalTrials.gov NCT00755573

## Introduction

The treatment of chronic pancreatitis patients can be a major clinical challenge. [1] Achieving control of pain, one of the main symptoms in this disease, can be difficult, and is often unsatisfactory for patients and doctors. [2] An evidence based approach to this pain for these patients does not exist, and to date there are no uniformly accepted guidelines for treatment.

One of the main factors contributing to this problem is the lack of evidence regarding the origin of chronic pancreatitis pain. [3] Complications of pancreatic inflammation such as dilated pancreatic duct, ductal stones and enlarged pancreatic head can be treated endoscopically or surgically, but numerous patients continue to suffer from pain despite technically successful interventions. [4], [5] Even bilateral splanchnicectomy, interrupting ascending nociceptive pathways, fails in a substantial number of patients. [6] In the last decade research has suggested that ongoing nociceptive input from the pancreas is not the only explanation for the debilitating abdominal pain in chronic pancreatitis. It is increasingly accepted that changes in central pain processing, e.g. central sensitization or a shift towards pro-nociceptive pain modulation, may result from chronic nociceptive input, manifest as spreading hyperalgesia.[7]–[9] Ultimately, this process may become entirely independent of nociceptive input and inhibitory pain modulation, leading to an autonomous pain state. [10].

Medication targeting altered central pain processing, e.g. gabapentinoids such as pregabalin, has been used successfully to treat other chronic pain disorders such as post-herpetic neuralgia and neuropathic pain of central origin.[11]–[13] In a recent publication, we demonstrated that pregabalin has a significant clinical analgesic effect in chronic pancreatitis patients. [14] Quantitative sensory testing (QST) is a useful tool to quantify pain processing in chronic pain patients, also in relation to the effectiveness of analgesic interventions. [15], [16] Apart from one recent study on S-ketamine for chronic pancreatitis pain and one using gabapentin for visceral pain in irritable bowel syndrome [12], [17], we are not aware of any studies having used QST to describe the influence of centrally active medication on pain processing in patients suffering from chronic pain disorders.

The aim of the present study is to evaluate the effect of pregabalin as adjuvant pain treatment on pain processing, measured by somatic QST, in patients with chronic pancreatitis. We hypothesized that the hyperalgesia in chronic pancreatitis patients with pain will undergo reduction under pregabalin treatment, but not under placebo treatment.

## Methods

### Study Overview

The protocol for this trial and supporting CONSORT checklist are available as supporting information; see Checklist S1 and Protocol S1.

This study was part of an investigator initiated double blind, placebo-controlled, parallel-group study of increasing doses of pregabalin conducted in the Netherlands (department of Surgery, Radboud University Nijmegen Medical Center) and Denmark (department of Gastroenterology & Hepatology, Aalborg Hospital, Aarhus University Hospital). [14] The study was approved by the responsible Ethical Committees in both countries (CMO region Arnhem-Nijmegen, Nijmegen, The Netherlands and The local Ethics Committee North Region, Aalborg, Denmark) and all patients provided written informed consent. This article presents a secondary and further analysis of the data obtained in a previous trial [14] focusing primarily on experimental (QST) endpoints.

### Patients

For trial inclusion, patients needed to have chronic abdominal pain typical for pancreatitis (i.e. dull epigastric pain more than 3 days per week for at least 3 months) and a diagnosis of chronic pancreatitis based on the Mayo Clinic diagnostic criteria. [18] Another inclusion criterion was the use of a stable regime of concomitant analgesic medication during the trial. Exclusion criteria were: painful conditions other than chronic pancreatitis, an abnormal electrocardiogram at screening visit, severe renal impairment, active (or history of) major depression, hypersensitivity to pregabalin or any of it components and pregnant or lactating patients. All patients that participated in this trial were included in this study and analyzed in an intention to treat analysis.

Clinical endpoints i.e. pain scores and side-effects of the main study are presented in more detail in the original manuscript. [14].

### Healthy Controls

A control group was recruited in Denmark for comparison with our chronic pancreatitis group to confirm the presence of spreading hyperalgesia at the baseline pre-medication measurement in the pancreatitis group. The controls were completely healthy and had no history of a medical condition that could interfere with our pain measurements.

### Randomization and Treatment

Eligible patients at our outpatient departments were randomly assigned in a one to one ratio to receive either pregabalin or placebo. A pseudo-random code was computer generated for the randomization blocks that had a size of six. Stratification of trial participants was based on the absence or presence of diabetes mellitus to minimize unbalance in distribution of undiagnosed diabetic polyneuropathy. Patients received increasing doses of either pregabalin or matching placebo for the study period of 3 weeks. Initial dose was 75 mg pregabalin twice a day (BID). After three days this was increased to 150 mg pregabalin BID, with a further increase to 300 mg BID after one week and for the rest of the study period. An equivalent regime was followed in the placebo arm. The same oral dosing schedule was prescribed to all patients. Daily dosages were split into two equivalent doses, one administered in the morning between 7 a.m. and 10 a.m. and one in the evening between 7 p.m. and 10 p.m. In the case of unacceptable side effects experienced by patients, a single downward dose titration was allowed. Patients had to stay on that final dosage for the remaining study period. Patients were instructed to taper their study medication after three weeks of treatment, by halving their dose for seven days, and then to stop medication. Patients and those administrating study medication, assessing outcomes, and analyzing data were blinded to group assignment.

### Study Visits

Patients considered for participation in this trial were screened for eligibility and physical fitness. A systematic physical examination including a neurological examination was performed to assess for any relevant conditions and neurological disorders. If eligible, they were randomized by their treating physician for placebo or pregabalin on their second visit, one week after their screening visit. During their second visit all patients had a baseline QST measurement, followed by another QST measurement at the end of the study period of three weeks, i.e. before they were instructed to taper their medication. During the whole study period patients were instructed not to change their daily pain medication. They were only allowed to take extra pain medication in the case of a painful exacerbation of their chronic pancreatitis.

### Quantitative Sensory Testing

QST took place using a standard temporal test sequence. [7] Testing in females was not standardized with regard to phase of the menstrual cycle because all female pancreatitis patients were amenorrhoeic. Both the examiners (one in Denmark and one in the Netherlands) were trained in and used the same QST protocol. They performed the measurements in the same way and setting. Pressure pain thresholds were based on two measurements and electrical pain thresholds were based on three measurements.

**Figure 1:**
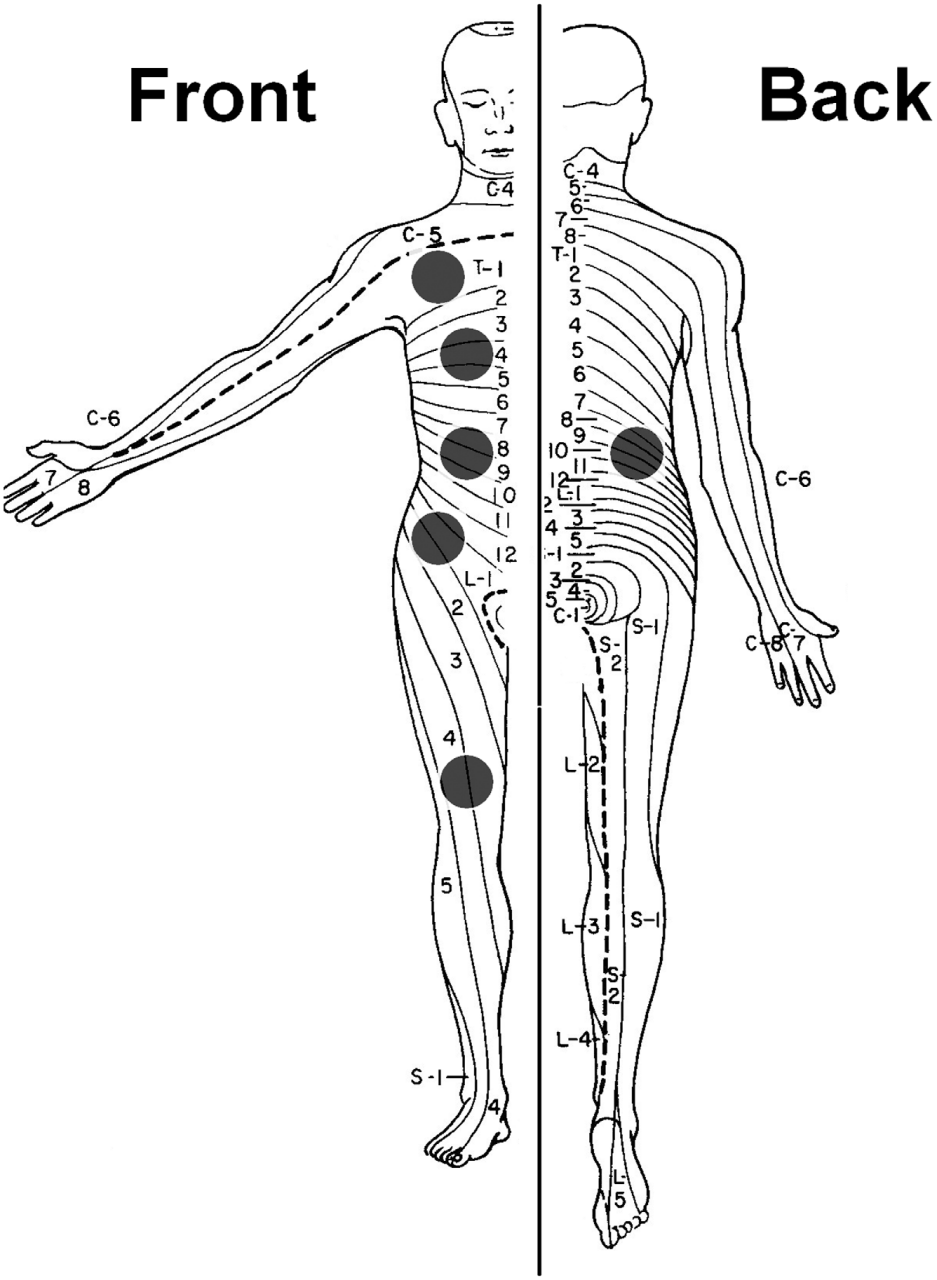
Dermatomes of measurement for quantitative sensory testing. Quantitative sensory testing was performed on the following sites on the dominant body side (black dots): clavicle (C5 dermatome), sternum (T4 dermatome), pancreatic site (dorsal and ventral T10 dermatome), hip region (L1 dermatome) and knee (L4 dermatome).

After initial QST training per participating subject, pressure pain thresholds were obtained for muscles overlying bone using a pressure algometer with a 1.0 cm^2^ probe (Somedic Sales AB, Horby, Sweden), at each of the following sites on the dominant body side: clavicle (C5 dermatome), sternum (T4 dermatome), pancreatic site (dorsal and ventral T10 dermatome), hip region (L1 dermatome) and knee (L4 dermatome) (Figure 1). The pancreas and more distant dermatomes were chosen to observe segmental and spreading hyperalgesia respectively. Two thresholds were measured: pressure pain detection threshold (pPDT) and pressure pain tolerance threshold (pPTT). As the primary endpoint, the sum of all the thresholds across dermatomes was calculated. [17].

Thresholds to electric constant current skin stimulation (Digistim; Biometer A/S, Copenhagen, Denmark; tetanic stimulation at 100 Hz, 0.2-ms square waves, self-adhesive electrodes 3 cm apart) were measured on the dominant side of the body at the same sites as for pressure pain thresholds. Two thresholds were measured: electric pain detection threshold (ePDT) and electric pain tolerance threshold (ePTT). As the primary endpoint, the sum of all the thresholds was again calculated. [17].

The conditioned pain modulation (CPM, previously known as DNIC (diffuse noxious inhibitory control)) paradigm was performed to test the ability of the patient to generate descending inhibitory modulation. [19], [20] Thus pressure pain thresholds (pPTT, the test stimulus) were determined before and after the cold pressor task (the conditioning stimulus), and the CPM effect was determined as the relative change (%) in pressure pain thresholds. For the cold pressor task the dominant hand was immersed in ice-chilled water (1.0°C ±0.3°C) continuously stirred by a pump. The patient was told to remove the hand from the water after two minutes of immersion - or sooner if the pain was considered to be intolerable – and the immersion time noted. Immediately after the cold pressor task, the subjects rated the pain experienced during the test by use of a visual analogue scale for quality control purposes. Pressure pain thresholds were obtained in the non-dominant L4 dermatome (knee) immediately before and after ice-water immersion.

### Outcome Measures

The primary effect parameter for the study was the between group difference (change) in sum of electric or pressure pain thresholds after three weeks of study medication vs. baseline values. [17] Between group differences in change in individual dermatome thresholds and CPM paradigm results were secondary endpoints.

### Statistical Analysis

A *pre hoc* power calculation based on QST as an endpoint was not performed because the study was a part of a randomized clinical trial that investigated pregabalin, powered for a clinical primary endpoint; i.e. change in clinical pain score.

For this mechanistic study we performed an intention to treat analysis. We performed statistical analysis using the Statistica for Windows Software Package (Release 7.0, Statsoft Inc, Tulsa, OK, USA). All baseline characteristics and measurements are given as medians with interquartile ranges. In view of the non-Gaussian data distribution purely non-parametric analysis was performed. Statistical significance was set at P≤0.05.

The sum of all dermatomes for electric and pressure pain detection and tolerance thresholds and the conditioned pain modulation results were compared between the control and the study group using Mann Whitney U testing to confirm spreading hyperalgesia and pro-nociceptive pain modulation shift in the pancreatitis patients. [14], [17].

We calculated differences (change) in sum of thresholds or individual thresholds between values at pre-medication baseline and after three weeks’ medication. We then compared these differences between the groups using Mann Whitney U testing. Further analysis consisted of comparison of placebo vs. pregabalin groups at pre-medication baseline and after three weeks’ treatment for the sum of thresholds, for individual thresholds, or for conditioned pain modulation values using Mann Whitney U testing.

## Results

### Enrollment and Baseline Characteristics

**Figure 2:**
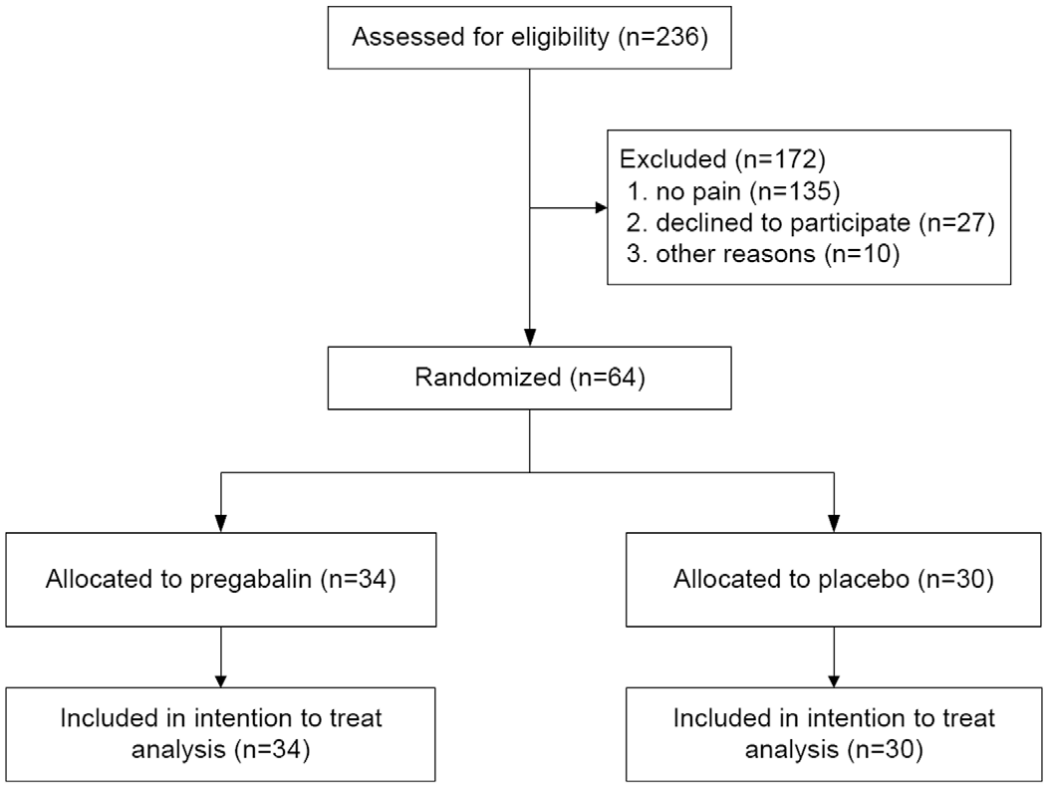
Study enrollment and randomization. The majority of patients not meeting inclusion criteria had passed away, were free of pain or were no longer being treated in either of the hospitals.

From October 2008 to May 2010 a total of 236 patients diagnosed with chronic pancreatitis in the last five years in one of both hospitals were screened and 64 patients were randomized; the study was completed without any incident. The majority of patients not meeting inclusion criteria were free of pain, had passed away or were no longer being treated in either of the hospitals. 64 patients completed the study and were finally analyzed in the intention to treat analysis (Figure 2). The number of patients randomized to pregabalin or placebo treatment was equally distributed between both hospitals. All patients (24 women, 40 men; median age 53 years (IQR (interquartile range) 45–62)) had pain due to chronic pancreatitis and were on a stable analgesic therapy. Their median opioid consumption was 60 mg (IQR 11–150) of morphine equivalents/day. Their median VAS score before start of trial medication was 4 (IQR 2–5) at rest and 5 (IQR 4–7) during activity. Demographic data of the placebo and pregabalin group are provided in Table 1. The healthy control group consisted of 15 volunteers (7 women, 8 men; median age 38 years (IQR 35–49)). Only age was significantly different between the healthy controls and chronic pancreatitis patients (P = 0.0001).

**Table 1:** Demographic and clinical characteristics of patients.

	Pregabalin (n = 34)	Placebo (n = 30)
Age (years)	52 (46–58)	55 (42–65)
Males - no. (%)	20 (59)	19 (63)
Etiology - no. (%)		
- Toxic-metabolic	16 (47)	17 (57)
- Idiopathic	11 (32)	11 (37)
- Genetic	2 (6)	0 (0)
- Autoimmune	1 (3)	0 (0)
- Recurrent and severe acute pancreatitis	2 (6)	1 (3)
- Obstructive	2 (6)	1 (3)
Diary pain score (visual analogue scale 0–10)		
- Average pain	4 (2–6)	3 (2–5)
- Maximal pain	6 (4–8)	5 (4–7)
Concomitant analgesics - no. (%)†		
- None	3 (9)	2 (7)
- Weak analgesics	7 (21)	11 (37)
- Strong analgesics	24 (71)	17 (57)
MEQ/day	80 (10–158)	49 (13–128)
Duration of chronic pancreatitis (months)	92 (55–132)	83 (60–147)
Diabetes mellitus - no. (%)	10 (29)	10 (33)
Previous interventions for chronic pancreatitis – no. (%)		
- Pancreas resection/drainage procedures	6 (18)	5 (17)
- Thoracoscopic splanchnic denervation	2 (6)	4 (13)
Patients treated with enzymes for pancreatic exocrine insufficiency - no. (%)	18 (53)	13 (43)
Ongoing alcohol abuse - no. (%)‡	7 (21)	11 (37)
Current smoker - no. (%)	26 (76)	22 (77)

All values are medians with interquartile ranges unless mentioned otherwise. Percentages may not total 100 due to rounding.

† Weak analgesics were defined as NSAIDS, paracetamol, codeine and tramadol. Strong analgesics were defined as opioid based therapies.

‡ Alcohol abusing patients were defined as female patients drinking >14 units of alcohol per week or male patients drinking >21 units of alcohol per week.

‘MEQ’ is morphine equivalents, ‘pregabalin’ is pregabalin study group and ‘placebo’ is placebo study group.

No statistical differences between groups were observed.

### Baseline Measurements

#### Pancreatitis vs. healthy controls

The sum for pressure and electric pain detection and tolerance thresholds of all dermatomes was significantly lower for the pancreatitis group at baseline vs. healthy controls (Table 2). At baseline chronic pancreatitis patients tolerated the cold pressor task for 35 seconds (IQR 24–70) and the healthy controls for 180 seconds (IQR 180–180), (P = 0.000004). The healthy control group exhibited a significantly greater CPM response than the pancreatitis patients (P = 0.008) (Table 2). These results confirm hyperalgesia and a shift to more pro-nociceptive pain modulation in our pancreatitis patients.

**Table 2:** Baseline data for conditioned pain modulation and sum of pain thresholds for pancreatitis patients vs. healthy controls.

	Pancreatitis	Control	P-value
SUM ePDT (mA)	28 (21–41)	47 (21–65)	0.026
SUM ePTT (mA)	44 (34–62)	68 (48–92)	0.017
SUM pPDT (kPa)	1912 (951–2551)	2285 (2018–3018)	0.008
SUM pPTT (kPa)	2694 (2110–3185)	3234 (2785–4018)	0.005
CPM (%)	4.2 (0.0–22.4 )	32.6 (10.4–41.8)	0.008

All values are medians with interquartile ranges. ‘Control’ is healthy control group and ‘Pancreatitis’ is chronic pancreatitis group. ‘ePDT’ is electric pain detection threshold, ‘ePTT’ is electric pain tolerance threshold, ‘pPDT’ is pressure pain detection threshold, ‘pPPT’ is pressure pain tolerance threshold and ‘CPM’ is conditioned pain modulation.

#### Pregabalin vs. placebo patients

The sum of all dermatomes for pressure and electric pain detection and tolerance thresholds at baseline was similar for the pregabalin vs. placebo groups (Table 3). The same applied to the individual dermatomal thresholds (Table 4).

**Table 3:** Sum of pain thresholds for pregabalin vs. placebo before and after treatment.

	Before		After	
	Pregabalin	Placebo	Pregabalin	Placebo
SUM ePDT (mA)	33.4 (23.6–43.5)	23.4 (18.9–33.8)	37.3 (27.9–51.6) •	26.1 (17.2–39.7)
SUM ePTT (mA)	53.4 (39.2–67.1)	41.7 (32.6–51.6)	52.3 (38.9–73.9)	44.0 (34.9–55.2)
SUM pPDT (kPa)	1936 (1063–2574)	1759 (902–2449)	1817 (1109–3312)	1817 (844–2585)
SUM pPTT (kPa)	2677 (2043–3136)	2720 (2307–3230)	2798 (2355–3945)	2853 (2131–3264)

All values are medians with interquartile ranges. •  =  Measurements after study treatment were significantly higher in the pregabalin group compared to the placebo group.

At baseline, patients in the placebo group tolerated the cold pressor task for 32 seconds (IQR 23–98) and in the pregabalin group for 40 seconds (IQR 23–60), this was not statistically different between groups. Also no significant difference was found in baseline CPM response between the placebo and pregabalin group (Table 4).

### Effects of Treatment - Change in Measurements after Three Weeks’ Study Medication

#### Electric pain thresholds

There was no significant difference in differences (change) in *sums* of electric pain detection and tolerance thresholds between the groups (Table 5).

**Table 4:** Conditioned pain modulation and pain thresholds for pregabalin vs. placebo before and after study treatment.

	Before		After	
	Pregabalin	Placebo	Pregabalin	Placebo
ePDT (mA)				
- C5	3.9 (2.5–5.1)	3.5 (2.4–6.0)	4.5 (3.4–5.6)	3.1 (2.1–6.0)
- T4	5.0 (3.0–7.0)	3.5 (2.7–6.3)	6.2 (4.2–8.5) •	3.9 (3.3–7.6)
- T10	5.8 (4.3–8.1)	3.7 (3.0–7.7)	6.5 (4.7–9.2)	4.9 (3.2–7.9)
- L1	4.8 (3.6–6.4)	4.3 (3.1–5.9)	6.6 (4.4–8.9)	4.7 (2.7–8.4)
- L4	5.7 (4.0–8.5)	4.1 (3.2–6.5)	5.8 (4.1–9.1) •	4.4 (3.2–6.6)
- T10 BACK	5.9 (3.8–7.2)	4.8 (3.8–6.9)	5.8 (4.3–10.3) •	4.3 (2.8–7.8)
ePTT (mA)				
- C5	6.9 (5.4–10.7)	5.4 (4.1–9.9)	9.6 (5.4–14.4) •	6.3 (5.0–10.7)
- T4	8.9 (5.6–13.0)	7.6 (5.1–10.7)	10.9 (7.5–16.2)	8.6 (5.6–11.6)
- T10	10.6 (6.1–12.7)	7.3 (3.7–10.7)	8.8 (6.1–15.1)	7.9 (5.6–12.6)
- L1	9.5 (6.5–13.5)	7.2 (5.2–11.1)	9.5 (7.0–14.8) •	7.1 (5.8–12.9)
- L4	9.9 (6.7–13.3)	7.4 (5.1–10.0)	9.1 (6.6–14.9)	7.2 (5.7–10.9)
- T10 BACK	11.7 (6.5–16.3)	9.3 (6.4–12.6)	9.1 (6.6–14.9)	8.9 (6.9–12.5)
pPDT (kPa)				
- C5	263 (142–334)	232 (106–380)	228 (130–321)	245 (115–398)
- T4	281 (195–392)	289 (139–409)	277 (169–421)	268 (147–359)
- T10	166 (97–302)	154 (85–264)	129 (65–328)	157 (61–306)
- L1	376 (207–511)	340 (197–571)	292 (207–566)	424 (168–528)
- L4	406 (235–601)	396 (176–613)	447 (177–689)	332 (204–641)
- T10 BACK	378 (211–474)	276 (162–522)	332 (132–549)	313 (161–480)
pPTT (kPa)				
- C5	421 (313–523)	378 (309–563)	451 (310–614)	459 (358–599)
- T4	481 (307–555)	422 (335–528)	431 (352–691)	371 (284–530)
- T10	246 (165–493)	257 (176–402)	280 (173–570)	236 (156–432)
- L1	578 (454–675)	548 (407–706)	551 (437–716)	581 (479–649)
- L4	608 (530–776)	614 (437–776)	733 (526–933)	700 (508–866)
- T10 BACK	574 (403–699)	561 (476–731)	612 (397–838)	537 (395–635)
CPM response (%)	0.9 (0.0–22.0)	8.9 (0.0–23.8)	0.0 (0.0–22.4)	0.0 (−4.0–19.8)

All values are medians with interquartile ranges. •  =  Measurements after study treatment were significantly higher in the pregabalin group compared to the placebo group.

**Table 5:** Change and percentage change in conditioned pain modulation and pain thresholds for pregabalin vs. placebo after study treatment.

	Pregabalin		Placebo	
	Change	Percentage change (%)	Change	Percentage change (%)
ePDT (mA)				
- C5	0.8 (0.2–1.9) •	24 (5–55)	−0.1 (−0.9–0.5)	2 (−25–16)
- T4	1.5 (−0.7–3.0)	39 (−13–83)	0.7 (−1.1–1.5)	17 (−16–49)
- T10	0.8 (−0.7–3.1)	12 (−11–52)	0.4 (−0.7–1.4)	8 (−9–40)
- L1	1.2 (−0.2–3.8)	25 (−6–71)	0.3 (−0.5–2.3)	11 (−11–51)
- L4	0.9 (−0.8–2.3)	14 (−16–46)	0.7 (−0.9–1.7)	18 (−22–37)
- T10 BACK	0.6 (−1.2–4.3)	16 (−22–75)	−0.6 (−1.4–1.3)	−14 (−29–14)
ePTT (mA)				
- C5	2.0 (−0.1–3.7) •	25 (−1–64)	0.7 (−2.4–1.9)	12 (−27–40)
- T4	2.6 (−0.6–5.1)	41 (−5–60)	1.5 (−0.9–2.9)	15 (−12–46)
- T10	1.0 (−0.8–2.4)	10 (−10–27)	0.8 (−1.2–2.4)	17 (−9–53)
- L1	1.9 (−0.8–4.9) •	29 (−7–59)	−0.2 (−2.3–2.1)	−3 (−28–38)
- L4	1.5 (−2.0–5.2)	22 (−20–53)	−0.3 (−1.2–3.0)	0 (−14–83)
- T10 BACK	1.1 (−1.9–4.8)	10 (−20–57)	−0.5 (−2.8–2.7)	−5 (−21–36)
pPDT (kPa)				
- C5	12 (−79–89)	7 (−39–34)	−13 (−51–50)	−7 (−19–31)
- T4	9 (−91–72)	4 (−34–26)	−3 (−89–45)	−1 (−29–13)
- T10	13 (−31–77)	6 (−31–53)	−1 (−51–26)	0 (−24–15)
- L1	63 (−137–117)	18 (−39–53)	12 (−99–135)	9 (−19–40)
- L4	80 (−129–176)	18 (−30–37)	0 (−50–88)	0 (−13–23)
- T10 BACK	41 (−63–89)	9 (−7–31)	34 (−73–90)	9 (−24–24)
pPTT (kPa)				
- C5	19 (−65–152)	3 (−17–40)	17 (−32–103)	5 (−9–31)
- T4	83 (−24–169) •	18 (−7–43)	−48 (−108–43)	−13 (−21–12)
- T10	10 (−56–111)	5 (−15–53)	20 (−103–56)	7 (−34–23)
- L1	−7 (−82–136)	−1 (−16–32)	69 (−149–144)	16 (−19–28)
- L4	17 (−253–278)	6 (−30–53)	75 (15–225)	12 (3–33)
- T10 BACK	41 (−74–194)	5 (−15–40)	14 (−165–81)	3 (−27–21)
SUM ePDT	6.0 (−1.2–15.0)	19 (−6–62)	2.3 (−1.9–5.8)	9 (−8–19)
SUM ePTT	7.6 (−7.1–13.5)	13 (−14–25)	2.7 (−7.1–10.9)	7 (−15–28)
SUM pPDT	311 (−155–526)	16 (−9–39)	131 (−330–329)	11 (−14–20)
SUM pPTT	226 (−265–593)	10 (−14–20)	193 (−192–380)	8 (−6–17)
CPM response (%)	1 (−4–18)	−18 (−100–134)	−2 (−23–6)	−100 (−136–16)

All values are medians with interquartile ranges. •  =  Measurements after study treatment were significantly higher in the pregabalin group compared to the placebo group.

For *individual* electric pain detection thresholds, difference (change) in dermatome C5 was significantly higher in the pregabalin group (0.8 vs. −0.1; P = 0.005), with a trend for T10 (P = 0.055) (Table 5).

For *individual* electric pain tolerance thresholds, threshold differences (change) for dermatome C5 (2.0 vs. 0.7; P = 0.04) and L1 (1.9 vs. −0.2; P = 0.05) were significantly higher in the pregabalin group (Table 5).

#### Pressure pain thresholds

There was no significant difference in differences (change) in *sums* of pressure pain detection and tolerance thresholds between the groups (Table 5).

There was no significant difference in differences (change) in *individual* dermatomal pressure pain detection thresholds between the groups (Table 5).

For *individual* pressure pain tolerance thresholds, threshold differences (change) for dermatome T4 (83 vs. −48; P = 0.004) were significantly higher in the pregabalin group (Table 5).

#### CPM response

The difference (change) in cold pressor task latency and CPM response was not significantly different between the study groups (Table 5).

### Effects of Treatment - Absolute Values after Three Weeks’ Study Medication

#### Electric pain thresholds

After 3 weeks’ study medication, *sum* of all dermatomes for electric pain detection thresholds was significantly higher in the pregabalin vs. placebo group (P = 0.01), but not for electric pain tolerance thresholds (Table 3).

For *individual* dermatomes, electric pain detection thresholds were significantly higher in the pregabalin group (T4; P = 0.04, L4; P = 0.05 and T10BACK; P = 0.05) (Table 4). For *individual* dermatomal electric pain tolerance thresholds, C5 (P = 0.05) and L1 (P = 0.03) were significantly higher in the pregabalin group.

#### Pressure pain thresholds

*Sums* of – or *individual* dermatome – pressure pain detection and tolerance thresholds were similar between groups (Table 3 and 4) after three weeks’ study medication.

#### CPM response

After three weeks of study medication patients in the placebo group tolerated the cold pressor task for 42 seconds (IQR 21–116) and in the pregabalin group for 46 seconds (IQR 27–77); this was not statistically significant. Also no significant difference could be found between groups for CPM response after study medication (Table 4).

## Discussion

Our study is the first to demonstrate that a three-week treatment with pregabalin in chronic pancreatitis patients results in a moderate antihyperalgesic effect compatible with a reduction of central sensitization. A shift toward more anti-nociceptive pain modulation appears less likely as mechanism due to the unaltered conditioned pain modulation (CPM) response. Interestingly, this early treatment effect is 1) visible only in dermatomes distant from the referred pancreatic area, and 2) more pronounced for electric skin thresholds than for pressure muscle thresholds. This implies 1) better effects on distant as compared to segmental central sensitization and 2) more effective hyperalgesia reduction in skin compared to deeper tissues. These results suggest that measuring pain sensitivity using quantitative sensory testing (QST) may prove useful in monitoring the effects of pain treatment in chronic pancreatitis and help us to diagnose and manage altered pain processing in chronic pain disorders.

Nociceptive input from the pancreas spreads via ascending pathways to spinal and supraspinal central nervous system structures in chronic pancreatitis. [21], [22] Ongoing nociceptive input increases neuronal excitability and synaptic strength, initially at the spinal level, a state characterized by hyperalgesia near the site of injury (segmental hyperalgesia). [3], [23] With persisting disease and nociception central sensitization spreads rostrally in the central nervous system. [8] This progression is more marked when descending inhibitory control mechanisms fail or in the presence of descending facilitation, and may in due course result in a widespread hyperalgesic state. [9] Ultimately, these central changes may become independent of peripheral nociceptive input, ending in an autonomous state. [6] Sensitization of the nervous system is not specific for chronic pancreatitis, but is common among other chronic pain disorders.[24]–[26] Congruently with the described course of events, chronic pancreatitis patients in our study did exhibit widespread hyperalgesia compared to healthy controls.

The treatment of pain in chronic pancreatitis patients is usually based on the WHO pain treatment ladder, which ends with opioid treatment. Opioids can provide effective analgesia in some pancreatitis patients, but may have considerable side effects or even induce hyperalgesia. [27] Recently, pain treatments more directly targeting the central nervous system, e.g. tricyclic antidepressants or gabapentinoids, have been introduced to better control disabling pain and hyperalgesia in chronic pain syndromes. [28] Particularly the use of gabapentinoids has shown clinical results in chronic pain disorders. [12], [29], [30] The clinical analgesic effect of pregabalin in chronic pancreatitis patients was recently published by our research group. [14] Two studies in an experimental pain model in healthy volunteers showed a reduction of hyperalgesia and central sensitization after gabapentin treatment. [31], [32] Only two studies, one with S-ketamine in chronic pancreatitis and one with pregabalin in irritable bowel syndrome, showed comparable reductions of hyperalgesia in patients treated with medication active in the central nervous system. [12], [17] To date a more prolonged reduction of somatic hyperalgesia and thus central sensitization has not been demonstrated in chronic pain patients in relation to pregabalin treatment.

In this study we failed to show a significant difference between groups regarding our primary outcome measure (change in sum of thresholds). The significant result regarding secondary outcome measure (change in individual dermatomal thresholds) suggests a moderate effect on spreading hyperalgesia. Interestingly, antihyperalgesic treatment with pregabalin resulted in a greater increase of electric pain thresholds than of pressure pain thresholds after three weeks treatment. A possible explanation is that pregabalin is initially more effective in reducing skin sensitization, as reflected by electric thresholds, as compared to deep tissue sensitization, as reflected by pressure thresholds. [32], [33] If this were true, one might expect greater decreases in deep tissue sensitivity with longer treatment periods in future studies.

In this study no significant improvement in conditioned pain modulation (CPM) could be found, suggesting that the main effect of pregabalin is to directly target central sensitization reflected by hyperalgesia, rather than the pro/anti-nociceptive balance of endogenous modulation. We did, however, demonstrate that before treatment, pancreatitis patients showed less inhibitory pain modulation than healthy controls in accordance with other studies. [9] However, it should be noted that CPM results exhibited considerable variability and are influenced by multiple factors. More research is clearly needed to define the relations between CPM, disease-related changes in central pain processing, and pain treatment effects. [9], [34].

A limitation of this study is the relatively small size of the chronic pancreatitis group. A larger sample of chronic pancreatitis patients would appear necessary to provide more detailed and significant evidence of the relation between pregabalin treatment, changes in pain scores and changes in hyperalgesia. While we did find parallel, separate reductions in pain scores and pain sensitivity in our patient collective, the study was not adequately powered to formally study – or prove – correlations between clinical pain reduction and reduction in hyperalgesia. Definitive proof of such a relationship awaits future larger and longer-lasting trials. It should be noted that most chronic pancreatitis studies are small due to the difficulties in recruiting large groups of uniform chronic pancreatitis patients. Better and larger national and international collaborations are necessary to permit larger and longer population based trials in chronic pancreatitis.

In this study an intention to treat analysis was performed conform the international standard for randomized clinical trials. It can be argued that for the mechanistic endpoints an analysis including only patients fully compliant with the study protocol should be performed (per protocol analysis). We therefore also performed a per protocol analysis, but we did not present these data in this manuscript, because there were no major differences compared to the intention to treat analysis. [14] We checked variability between the study groups and found that standard deviations were comparable between both the groups at the different times. The presence of diabetes mellitus, alcohol consumption and the wide range of morphine dosages might have influenced our results. Certainly some patients might have shown diabetic polyneuropathy or morphine induced hyperalgesia, which could have biased our results. However during the physical examination of trial participants no peripheral sensory or motor disturbances were detected and all baseline characteristics were equally distributed between both treatment groups. There was a significant difference in age between the healthy controls and the chronic pancreatitis patients. The importance of this difference is difficult to assess. Some studies described an increase of pain thresholds during aging [35], others showed no effect [36] and some showed a decrease in thresholds during aging [37].

Another limitation of the study is the absence of a long-term follow-up. We only measured effects after a relatively short treatment period of three weeks. At this time, modest reductions in distant skin hyperalgesia were already noticeable and significant, albeit without return to normal values (i.e. as in healthy volunteers). This reduction in hyperalgesia occurred in a patient population with a long history of chronic pancreatitis, generally regarded as being particularly difficult to manage.

In conclusion our study provides first evidence that pregabalin modestly reduces the spreading hyperalgesia as manifestation of central sensitization associated with chronic pancreatitis pain. This effect was evident after three weeks of pregabalin treatment and was most evident for electric skin pain thresholds. However more research is needed to predict the long-term effects and define effective dosage schemes for pregabalin use in different stages of chronic pancreatitis.

## Supporting Information

Checklist S1 **CONSORT checklist.**
(DOC)

Protocol S1 **Trial protocol.**
(DOC)
