# Supplementary material for: Effects of Pregabalin on Central Sensitization in Patients with Chronic Pancreatitis in a Randomized, Controlled Trial
Source: PLoS One. 2012 Aug 6;7(8):e42096. doi: 10.1371/journal.pone.0042096 (PMC3412837; doi:10.1371/journal.pone.0042096)
Supplement: Protocol S1 — Trial protocol. (DOC) [file pone.0042096.s002.doc]

# PROTOCOL

# A randomized, double-blind, placebo-controlled, PARALLEL GROUP clinical Study OF PREGABALIN in patients with chronic pancreatitis

**Department of Gastroenterology, Aalborg hospital**

**Aalborg, Denmark, 9000**

**Departments of Surgery and Anaesthesiology**

**UMC St. Radboud**

**POB 9101, 6500 HB Nijmegen**

**EXPECTED DATE OF BEGINNG: 01.08.2009**

**EXPECTED DATE OF TERMINATION: 01.08.2011**

**STUDY DURATION: 24 MONTHS**

PROTOCOL SIGNATURE PAGE

**PRINCIPAL INVESTIGATORS**

**Asbjørn M Drewes, MD, PhD, Aalborg hospital**

**Harry van Goor, MD, PhD, UMC St Radboud**

**Oliver H.G. Wilder-Smith, MD, PhD, UMC St radboud**

**Date**

|  |  |
| --- | --- |

###### SUB INVESTIGATOR Stefan Bouwense Date

|  |  |
| --- | --- |

**The signatures of the principal investigators and the sub investigator, indicate that they have read and understand the protocol and agree to conduct the study in accordance with the protocol, Good Clinical Practices, and International Conference on Harmonization.**

**SIGNATURE HEAD OF DEPARTMENT**

**Prof. Dr. C.J.H.M. van Laarhoven**

**Date**

|  |  |
| --- | --- |

**PARTICIPANTS:**

**PRINCIPAL INVESTIGATORS**

Harry van Goor, MD, PhD, Oliver H.G. Wilder-Smith, MD, PhD

Departments of Surgery and Anaesthesiology

Radboud University Nijmegen Medical Centre

POB 9101, 6500 HB Nijmegen

The Netherlands

Telephone No. +431 24 366 8086

**SUBINVESTIGATOR**

Stefan Bouwense

Departments of Surgery and Anaesthesiology

Radboud University Nijmegen Medical Centre

POB 9101, 6500 HB Nijmegen

The Netherlands

Telephone No. +431 24 366 8086

**STUDY CENTRE**

Department of General Surgery Radboud University Nijmegen Medical Centre POB 9101
6500 HB Nijmegen
The Netherlands

**DRUG SUPPLIES AND FINANCIAL SUPPORT**

Pfizer Inc. Clinical R & D (Gastroenterology / Hepatology) Global Research and Development Sandwich, Kent. United Kingdom

List of Abbreviations

The following abbreviations and special terms are used in this study protocol.

| Abbreviation or special term | Explanation |
| --- | --- |
| AE | Adverse event |
| AUC | Area under the curve |
| BP | Blood pressure |
| BID | Two times daily |
| CRF | Case report form |
| ECG | Electrocardiogram |
| EEG | Electroencephalography |
| Ethics Committee | Synonymous to Institutional Review Board and Independent Ethics Committee |
| GCP | Good Clinical Practice |
| CGRP | Calcitonin-gene related peptide |
| ICH | International Conference on Harmonisation |
| IRB | Institutional Review Board |
| NGF | Nerve growth factor |
| mBPI-sf | Modified brief pain inventory-short form |
| OAE | Other Significant Adverse Event |
| QOLQ | Quality of life Questionnaire |
| PDQ | Pain DETECT questionnaire |
| PGIC | Patient Global Impression of Change |
| PDT | Pain detection threshold |
| PK | Pharmako kinetic |
| Principal investigator | A person responsible for the conduct of a clinical study at a study site. Every study centre has a principal investigator. |
| SAE | Serious adverse event |
| SD | Standard deviation |
| SUSAR | Suspected Unexpected Serious Adverse Reaction |
| SP | Substance P |
| VAS | Visual analogue scale |
| WHO | World Health Organisation |

**SCHEDULE OF ACTIVITIES**

| **Protocol Activity** | Screen  **Day**  **-28 to -7** | Day 1 | Day 4 | Day 7 | Day 11 | Day 14 | Day 17 | End of Treatment and follow up  **Day 21 + 14 ^j^** | Day 21 + 14 and 7 days forward |
| --- | --- | --- | --- | --- | --- | --- | --- | --- | --- |
| Informed Consent | X |  |  |  |  |  |  |  |  |
| Specific Medical History and Physical Examination | X |  |  |  |  |  |  |  |  |
| General Medical History and Physical Examination | X |  |  |  |  |  |  |  |  |
| Weight | X |  |  |  |  |  |  | X |  |
| Laboratory |  |  |  |  |  |  |  |  |  |
| Haematology | X |  |  |  |  |  |  | X |  |
| Blood Chemistry | X |  |  |  |  |  |  | X |  |
| Urinalysis | X |  |  |  |  |  |  | X |  |
| Coagulation | X |  |  |  |  |  |  | X |  |
| Pregnancy test ^a^ | X |  |  |  |  |  |  | X |  |
| ECG | X |  |  |  |  |  |  | X |  |
| Registration/Randomization | X |  |  |  |  |  |  |  |  |
| Study Treatment |  |  |  |  |  |  |  |  |  |
| Dosing pregabalin ^c^ |  | X --------------------------------------------------------------------------------- X | | | | | | | |
| Assessments |  |  |  |  |  |  |  |  |  |
| Skin | X | X |  |  |  |  |  | X |  |
| Muscle | X | X |  |  |  |  |  | X |  |
| Cold pressor test | X^d^ | X |  |  |  |  |  | X |  |
| Concomitant Medication ^e^ | X |  |  |  |  |  |  | X |  |
| Questionnaires |  |  |  |  |  |  |  |  |  |
| QOLQ ^f^ | X |  |  |  |  |  |  | X |  |
| mBPI-sf ^g^ | X |  |  |  |  |  |  | X |  |
| PD-Q ^h^ | X |  |  | X |  |  |  | X |  |
| PGIC ^i^ | X |  |  | X |  |  | X | X |  |
| Patient pain diary | X | X | X | X | X | X | X | X |  |
| Interview |  |  | X | X | X | X | X | X |  |

^a^ Pregnancy tests will be done in females not postmenopausal for more than 1 year or sterilised.

^c^ 75mg BID Day 1-3, 150mg BID day 4 – 7, 300mg BID Day 8 – 21 + 14 days followed by a 1 week medication taper, on half their maximum tolerated dose

^d^ Cold pressor test training

^e^ Concomitant medications will be assessed and noted at screening. Randomized patients will be advised to maintain their usual medication. As needs pain medication will be recorded in the diary.

^f^ Quality of life Questionnaire

^g^ Modified brief pain inventory-short form

^h^ Pain DETECT questionnaire

^i^ Patient Global Impression of Change. The PGIC questionnaire will be administered during outpatient clinic visits on Days 1 and Day 21+ 14 days and by telephone on days 7, and 17.

^j^ 21+14: the study treatment will continue for optimally 21 days with possibility of extending the study up to 14 days for logistical purposes, therefore the trial medication will be packed to cover 35 days and the patients will return excess trial medication at the last visit or the next visit in the outpatient clinic.

TABLE OF CONTENTS

1. INTRODUCTION 7

1.1. Indication 7

1.2. Background and Rationale 7

1.2.1. Chronic Pancreatitis 7

1.2.2. Clinical versus Experimental Pain 7

1.2.3. Treatment of Neuropathic Pain with Pregabalin 8

1.2.4. Neuropathic Pain and Chronic Pancreatitis 9

2. STUDY OBJECTIVES AND ENDPOINTS 9

2.1. Objectives 9

2.2. Endpoints 10

2.2.1. Primary Endpoints 10

2.2.2. Secondary Endpoints 10

3. STUDY DESIGN 10

4. SUBJECT SELECTION 10

4.1. Inclusion Criteria 11

4.2. Exclusion Criteria 11

4.3. Randomization Criteria 12

5. STUDY TREATMENTS 12

5.1. Drug Supplies 13

5.2. Concomitant Medication(s) 13

6. STUDY PROCEDURES 13

6.1. Screening 13

6.2. Study Period 14

6.3. Subject Withdrawal 15

7. ASSESSMENTS 16

7.1. Visual Analogue Scale (VAS) 16

7.2. Skin and Muscle Stimulation 16

7.2.1. Tetanic Electrical Skin Stimulation 16

7.2.2. Deep Muscle Stimulation 16

7.3. Cold Pressor Test in Combination with Pressure Stimulation 17

8. ADVERSE EVENT REPORTING 17

9. DATA ANALYSIS/STATISTICAL METHODS 20

10. DATA HANDLING AND RECORD KEEPING……………………..............................21

11. ETHICS 21

12. Financial support and Insurance..................................................................22

13. PUBLICATION OF STUDY RESULTS 22

14. REFERENCES 23

INTRODUCTION

## Indication

Chronic Pancreatitis

## Background and Rationale

### Chronic Pancreatitis

The aetiology of pain in chronic pancreatitis remains to be elucidated. Therefore no common guidelines for the management of the pain exist and it is a topic of great discussion. The area is further complicated by the fact that only a few clinical trials have been carried out (Wilder-Smith *et al.* 1999 [1]). The best attempt to establish clear guidelines for the treatment of chronic pancreatitis is “American Gastroenterological Association Medical Position Statement: Treatment of Pain in Chronic Pancreatitis” (Warshaw *et al.* 1998 [2]). Initial treatment consists of low fat diet and non-narcotic analgesics, which can be supplemented by oral pancreatic enzymes and proton pump inhibitors. If an acceptable level of pain relief is not obtained with these drugs, only opioids remain for the management of pain. Opioids have a number of well-known adverse effects including elevation of smooth muscle tone (affecting gastrointestinal motility), toxicity in the central nervous system and especially induction of addiction. Many patients suffering from chronic pancreatitis have a history of alcoholic abuse making opioids, with their associated abuse potential, less suitable for these patients. Alternatives to medical treatment exist in the form of nerve blockade, lithotripsy and surgical treatment. However, results from studies of non-medical treatment modalities are equivocal and medical analgesic therapy must still be considered as the first choice in the management of painful chronic pancreatitis. Thus the importance of identifying potential new treatment regimes for the treatment of pain in chronic pancreatitis is clear.

### Clinical versus Experimental pain

Basic mechanisms in pain perception, transduction and processing can be explored by means of human experimental pain models. These models, when applied to healthy volunteers or to patients, provide an important translational link between preclinical animal testing and human clinical trials. In clear contrast to clinical pain, experimental pain models allow the possibility of controlling the duration, the intensity and the nature of the pain stimulus. Thanks to the exact control of the stimuli, the effects of analgesics can be assessed by performing standardized tests before and after analgesic administration. As pain is a multidimensional perception it is obvious that the reaction to a single stimulus of a certain modality only represents a limited part of the pain experience and therefore a variety of stimulus modalities and methods for pain assessment are required. Analgesics may differ in their effectiveness, influenced by both the nature of the stimulus and the tissue under investigation. This explains the importance of testing different tissues with multimodal pain models. By these methods, the most comprehensive and differentiated information about the analgesic of interest is obtained.

In the present study we have chosen to stimulate the skin and muscle in patients with chronic pancreatitis to elucidate changes in central pain transduction and processing. It has been shown previously that patients with chronic pancreatitis have hypoalgesia to visceral stimulation and this was found to be true for both the duodenum and the oesophagus. Further, an increased size of the referred pain area was found for the patients compared to healthy controls (Dimcevski *et al*. 2006 [3]; Drewes *et al*. 2006 [4]). The hypoalgesia to direct stimulation may reflect increased activity in descending control systems capable of dampening the peripheral input, whereas the increase in the somatic referred pain area to the same visceral stimulations probably reflects central hyperexcitability. Well established and validated models for multimodal stimulation of rectum exist (Drewes *et al*. 2002 [5] & 2003a [7]).

Recently an experimental pain model has been used to compare the effect of oxycodone (thought to work at the κ-opioid receptor in the peripheral visceral afferents) and morphine in equipotent doses (Staahl *et al.* 2006 [6]). The model compared the effect of the opioids on multimodal pain stimulations in the skin, muscle and esophagus. The study showed that oxycodone attenuated visceral pain better than morphine (and placebo) and hence elucidated a new aspect of visceral pain treatment that could never have been found in conventional phase 3 studies. In another recent study we showed that gabapentin was effective in attenuating deep muscle pain, a finding that is consistent with the wide use of this drug in various complex pain syndromes (Arendt-Nielsen *et al*. 2007 [8]).

### Treatment of neuropathic pain with pregabalin

Central sensitization is an essential factor in the development of neuropathic pain (Baron 2001 [9], Johnson *et al.* 2001 [10]) and in animal experiments the NMDA receptor has been shown to be involved in this process. Calcium plays a central role, as an increased influx of calcium into the neuron is observed when the NMDA receptor is activated. The enhanced concentration of intracellular calcium functions as a second messenger for a number of neurotransmitters and calcium thus contributes to the maintenance of the central sensitization (Nicholson 2000 [11]).

Pregabalin exerts a range of effects in pain transmission and although the precise mechanism of action is not completely understood, it likely involves the binding of the drug to calcium channels in the central nervous system (Ben-Menachem 2004 [12]). In animal experiments it has been shown that pregabalin primarily exerts its effect in the dorsal horn, where a reduced pain signal is seen. Pregabalin is a ligand of the α_2_-δ subunit of the voltage-gated calcium channel and binding of pregabalin to this site results in reduced calcium influx at the nerve terminals and therefore a reduced release of several excitatory neurotransmitters, including glutamate, substance P (SP), calcitonin-gene related peptide (CGRP) and noradrenaline. This may result in inhibition or reduction of the sensitization mentioned above. Moreover, this may be the basis for the analgesic effect. It has been shown, in animal experiments, that binding of pregabalin to calcium channels is able to counteract central sensitization in the dorsal horn.

The evidence for the clinical effect of pregabalin in neuropathic pain is substantial and is documented in a number of randomized clinical trials (Dworkin *et al.* 2003 [13], Sabatowski *et al.* 2004 [14], Rosenstock *et al.* 2004 [15]). The recommended dose for treating neuropatic pain is between 150 mg to 600 mg daily, which is similar to the dose chosen for this study.

### Neuropathic pain and chronic pancreatitis

In patients with chronic pancreatitis the pancreatic nerves have been found to have a greater diameter and the area innervated by a single nerve is smaller (Bockman *et al.* 1988 [16]). A neural genesis of pain therefore seems likely. The basis of the neurogenic generation of pain may be due to an altered expression of neuropeptides like SP and CGRP. The concentration of nerve growth factor (NGF) and its receptor TrkA is increased in inflammatory areas which may lead to an enhanced transcription of SP and CGRP, both of which are transmitters in the pain system (Di Sebatiano *et al.* 2003 [17]). Interestingly, the release of SP is known to be reduced by agents such as pregabalin.

Support for a neuropathic component of the pain of chronic pancreatitis is also found in clinical observations, where the pain is typically described as largely constant background pain with shooting, burning and lancinating episodes that may mimic that seen in peripheral neuropathies. Finally, evidence for central neuroplastic findings and strong descending inhibition, which may reflect the pain mechanisms in neuropathic pain have been found in recent studies (Dimcevski *et al.* 2006 and 2007 [18, 19]). Thus neuropathic and “true” visceral pain components appear to play a role in pancreatic pain. Hence the potential to extrapolate from clinical trials demonstrating the activity of pregabalin in the treatment of neuropathic pain to the treatment of the possible neuropathic component of chronic pancreatitis is of great interest.

# STUDY OBJECTIVES AND ENDPOINTS

The aim of the study is to investigate the effect of pregabalin in pain resulting from chronic pancreatitis. The effect will be investigated by means of questionnaires concerning the daily experience of pain and the general quality of life. Furthermore the patients will be invited to participate in a programme of human experimental pain testing (quantitative sensory testing = QST). The results from human experimental pain testing may help us to understand the mechanisms of action of pregabalin in this patient population.

## Objectives

The present study consists of two parts: one clinical and one involving human experimental pain testing (i.e. QST). In the clinical part the aim is to investigate the effect of pregabalin on clinical pain resulting from chronic pancreatitis. In the clinical part of the study, the primary objective is to understand the effect of pregabalin on the daily pain experienced by patients with chronic pancreatitis.

The experimental part aims at evaluating the effect of pregabalin using QST in controlled and standardized circumstances. In the experimental part of the study the primary objective is to understand the effect of pregabalin on altered pain processing in patients with chronic pancreatitis.

In the clinical part of the study a secondary objective is to understand the effect of pregabalin on the quality of life of patients with chronic pancreatitis. Further secondary objectives are to understand the safety and tolerability of pregabalin in this patient population.

## Endpoints

### Primary Endpoints

The primary endpoint for the study is analgesic effect of pregabalin therapy.

In the *clinical part* of the study, the primary efficacy parameter for analgesic effect is pain relief. This is defined as the reduction in VAS pain scores at the end of the study (day 21) as compared to the pre-treatment level. This effect is further assessed as changes in the daily experience of pain, which will be assessed using questionnaires, including the modified brief pain inventory-short form (mBPI-sf), the pain*DETECT* questionnaire (PD-Q, Freynhagen *et al*. 2006 [21]) and patient global impression of change (PGIC, Farrar *et al*. 2001 [22]).

In the *experimental part*, the primary endpoint for analgesic effect is change in pain processing. This is defined as the reduction pain tolerance thresholds in the dermatomes at the end of the study (day 21) as compared to the pre-treatment level. Pain processing will be assessed using QST parameters. All changes are compared to baseline recordings.

### Secondary Endpoints

Secondary efficacy parameters are changes in quality of life compared to baseline level. The European Organization for research and treatment of cancer quality of life questionnaire (QOLQ, Bloechle *et al.* 1995 [23], will be used. Also the tolerability of the drug in this patient population will be compared to placebo.

In the experimental part of the study the difference of the QST stimulus are considered secondary efficacy parameters. The experimental baseline recordings will also be compared to published normal values from healthy controls to evaluate general aspects of pain processing in the patient group.

# STUDY DESIGN

This will be a randomized, double-blind, parallel group, placebo-controlled study to investigate the effects of pregabalin on clinical and experimental pain in 60 patients with chronic pancreatitis (30 in Denmark and 30 in the Netherlands).

Participation in the study will involve three outpatient clinic visits, including the screening visit. The second and third outpatient clinic visit will include skin and muscle sensitivity testing. In addition, all subjects will undertake five telephone interviews.

During the study, the patients will be randomized to receive placebo or pregabalin (Lyrica®). The maximum daily dose of pregabalin is 300 mg administered twice per day. Pregabalin is titrated in 2 steps: for the first 3 days 75mg BID is administered. From Day 4 to Day 7, 150 mg BID is administered. From Day 8 to Day 21, 300 mg BID is administered. At the end of the study, patients will undertake a medication taper and will take half their maximum tolerated daily dose of pregabalin for 7 days and then discontinue dosing.

# SUBJECT SELECTION

The investigator will screen sufficient patients to allow 60 patients to complete the study.

## Inclusion Criteria

# Male or female patients between the ages of 18 and 70 years with a diagnosis of chronic pancreatitis, diagnosed using the Marseille-Rome/Cambridge diagnostic criteria (Etemad and Whitcomb, 2001 [24]). Both diabetic and non-diabetic patients will be allowed to enter the study.

### The patients must suffer from chronic abdominal pain typical for pancreatitis, meet the criteria for chronic pain (pain ≥ 3 days per week in at least 3 months) and must consider their pain as severe enough for medical treatment.

# Personally signed and dated informed consent document indicating that the patient has been informed of all pertinent aspects of the trial.

# Patients willing and able to comply with the scheduled visits, treatment plan, laboratory tests and other trial procedures.

## Exclusion Criteria

1. Patients with evidence or history of medical or surgical disease of importance for this study as judged by investigator
2. Presence or history of major depression
3. Patients with previously diagnosed moderate to severe renal impairment. Patients with creatinine values > 2x ULN and/or with a significant change to their normal values should be excluded.
4. Patients with a screening 12-lead ECG demonstrating any of the following: heart rate >100 bpm, QRS duration >120 msec, QTc interval >450 msec, PR interval >210 msec, any clinically significant rhythm abnormality, any evidence of myocardial ischemia or injury.
5. Patients with any clinically significant laboratory abnormalities that in the opinion of the investigator may increase the risk associated with trial participation or may interfere with the interpretation of the trial results.
6. Patients treated with pregabalin (Lyrica®) during the previous 4 months.
7. Treatment with an investigational drug within 4 months preceding the first dose of study medication of importance for this study as judged by investigator.
8. Female patients who are pregnant or lactating, or intend to become pregnant. Male patients who intend to father a child during the course of the study. A pregnancy test will be conducted at visit 1 and 3 to ensure that female patients are not pregnant during the study period.
9. Patients unwilling or unable to comply with the lifestyle guidelines.
10. Patients must not suffer from painful conditions other than chronic pancreatitis.
11. Clinical significant illness within two weeks of participating in this study.
12. Involved in planning or conducting the study.
13. Hypersensitivity to pregabalin or any of its components.

## Randomization Criteria

Subjects will be randomized to the study providing they fulfill all of the entry criteria at screening. A computer-generated pseudo-random code will be used to assign subjects to treatment. For the groups there will be 2 randomization lists; one for diabetic patients and one for non-diabetic patients. This is to ensure an even distribution of diabetic and non diabetic patients in the placebo group and the pregabalin group. Individual treatment codes in sealed envelopes, indicating the treatment randomization for each randomized patient, will be available to the investigator(s) at the study centre. The individual treatment codes must not be broken except in medical emergencies when the appropriate management of the patient necessitates knowledge of the treatment randomization.

# STUDY TREATMENTS

Patients will take either placebo or pregabalin, 75mg BID for the first 3 days, 150 mg BID for the next 4 days followed by 300 mg BID from Day 8 to Day 21 +14 days. If a patient does not tolerate 300mg BID it will be acceptable for them to maintain 150mg BID for the remainder of the study, following discussion and guidance from the investigator.

Pregabalin and placebo will be presented as grey capsules, which should be taken with approximately 250 ml water.

In order to maintain the blind, all patients will take doses of drug or placebo twice a day. Doses should be taken between 07:00 and 10:00 and between 19:00 and 22:00 (i.e. at approximately 12 hourly intervals).

After the last day of the study, the patients will taper their medication as detailed below:

Taper medication will be dispensed together with all other study medication at Visit 2. Taper will be administered to subjects who discontinue the study early only if they have completed 4 or more days of study drug. Thus patients who are unable to escalate from 75mg BID will simply cease medication. For all other subjects, the tapering period is 7 days.

- Subjects who were taking 300 mg BID pregabalin will have their dose reduced to 150 mg BID.
- Subjects who were taking 150 mg BID pregabalin will receive 75 mg BID.

Subjects who were taking placebo will continue on matching placebo for 7 days.

The amount of investigational products that is dispensed and returned will be recorded in the patient specific Case Report Form (CRF) by the investigator(s). It is essential that designated study personnel account for all medication. All discrepancies between amounts of investigational products dispensed and returned must be recorded and explained.

## Drug Supplies

Supplies will be provided in bottles by Pfizer Clinical Research Operations.

- Pregabalin capsules orally 75 mg (total dose of 150 mg/day)
- Pregabalin capsules orally 150 mg (total dose of 300 mg/day and 600mg/day)
- Matching placebo capsules orally.

The study drugs will be packed and labeled under the responsibility of Pfizer Clinical Research Operations.

## Concomitant Medication(s)

Patients will be instructed not to change their regular pain treatment during the trial period, this will be recorded twice, at the screening visit and the last visit. Additional pain medication (taken as needed) will be used and recorded at the telephone interviews and in the diary.

# STUDY PROCEDURES

The pain intensity, neuropathic pain component, global well-being and quality of life are tested with standardized questionnaires. The study will take place in Radboud University Nijmegen Medical Centre.

## Screening

Visit 1; Screening (28 – 7 days before the first dose of study drug):

- Obtain informed consent.
- Physical examination.
- General and specific medical history (including drug/alcohol/tobacco consumption).
- Concomitant medication.
- Questionnaires (QOLQ, mBPI-sf, PD-Q and PGIC) are filled out.
- Blood and urine for hematology, clinical chemistry and urinalysis.
- Pregnancy test (urine) will be done in females not postmenopausal for more than 1 year or not sterile.
- 12-lead ECG.
- Blood pressure and pulse rate (supine and standing). Supine BP/PR should be measured following resting for 5 minutes. Standing BP/PR should be measured following standing for 2 minutes.
- Diary instruction. The patient will start completing the pain diary 7 days prior to the first dose of study drug.
- Experimental testing (training): pressure and electrical stimulation (skin and muscle) as described below.
- Cold pressor test (training) as described below.

Patients fulfilling inclusion and exclusion criteria are randomized and invited to join the study.

The duration time of the first visit will be approximately 4 hours.

## Study Period

Seven days prior to the first dose of study drug, the patients will start to score pain levels in a pain diary. The average pain over the previous 24 hours and the worst pain over the previous 24 hours will be assessed using the VAS score. Usage of pain medication will also be recorded in the diary

Visit 2:

Day 1, first dose of study drug:

The patients visit the clinic and are instructed to start the trial medication if laboratory tests are normal. Ahead of the first dose of study drug the patients will undergo multimodal testing. They will have the skin in segments C5, T4, T10, L1 and L4 stimulated electrically. Also deep muscle pain will be assessed on the same places, using pressure stimulation. A cold pressor test is performed on the right hand and the deep muscle pain is measured on the left thigh both before and after the cold pressor test. Pain thresholds will be assessed using visual analogue scales (VAS).

These assessments are done before starting the treatment on Day 1 and on Day 21 + 14 days.

Patients will start the trial medication and score pain levels in the diary with a VAS value for the average pain over the previous 24 hours and a second VAS value for the worst pain over the previous 24 hours. Use of as needs pain medication will also be recorded in the diary.

The duration time of the second visit will be approximately 2 hours.

Telephone interviews

On days 4, 7, 11, 14 and 17 a study nurse or doctor will call the patients to assess the severity and tolerability of any adverse events.

On Day 7 a study nurse or doctor will call the patients to assess the adverse events and to administer the PD-Q and PGIC questionnaires. The dose of study drug will be discussed. Patients with acceptable adverse events will increase the dose to 300 mg BID. Patients experiencing unacceptable adverse events on 150 mg BID will be taken out of the study and will be replaced.

Visit 2:

All patients will visit the centre on day 21 + 14 days.

- Physical examination.
- Concomitant medication
- Questionnaires (QOLQ, mBPI-sf, PD-Q and PGIC) are filled out.
- Blood and urine for haematology, clinical chemistry and urinalysis.
- Pregnancy test (urine) will be done in females not postmenopausal for more than 1 year or not sterile.
- Experimental testing as described in visit 1: pressure and electrical stimulation (skin and muscle).
- Cold pressor test.

Patients will be instructed to taper their study medication by halving their study drug dose for 7 days and then stopping the medication.

The duration time of the third visit will be approximately 3 hours.

## Subject Withdrawal

If unacceptable adverse effects are experienced by the patient between the telephone calls, the patient should contact the investigator. Based on a medical judgment, the dose of study drug may be reduced and the treatment continued or the patient may be withdrawn from the study. Follow up consultations and eventually additional blood tests will be arranged as by request judged by the investigator. The minimum end dose will be 150 mg BID, otherwise the subject will be withdrawn. Withdrawn patients or dropouts will be replaced until a number of 60 patients have fulfilled the study.

# ASSESSMENTS

## Visual Analogue Scale (VAS)

Only one scale will be used in both the clinical and experimental studies to secure comprehensible and uniform recordings. A scale that has previously been shown to be reliable and robust to discriminate sensations in somatic and visceral pain will be used (Drewes *et al.* 2003c [26]). This VAS will be used to assess both non-painful and painful sensations in the skin and muscle.

It is presented as a 10-cm line, anchored by verbal descriptors: The intensity of the *non-painful sensations* will be scored with the following descriptors added to facilitate the scoring: 1 – vague perception of mild sensation; 2 – definite perception of mild sensation; vague perception of moderate sensation; 4 – definite perception of moderate perception. For the *painful sensations* the patients will use the scale from 5 to 10 anchored at 5 – pain detection threshold (denoted PDT); 6 – slight pain; 7 – moderate pain threshold (denoted MPT); 8 – medium pain intensity; 9 – intense pain and 10 – unbearable pain. This part of the scale is colored red for a clear visual separation of the non-painful and painful range of sensations.

The score is measured from the zero-anchor to the patient’s mark. Using a millimeter scale to measure the patient's score will provide 101 levels of pain intensity.

This VAS scale will also form the pain assessment in the patient diary.

## Skin and muscle stimulation

### Tetanic electrical skin stimulation

Thresholds to electrical constant current skin stimulation (Digistim®; Biometer A/S, Copenhagen, Denmark; tetanic stimulation at 100Hz, 0.2ms square waves, self-adhesive electrodes 3cm apart) will be measured at the following left-sided sites: thorax (T4 dermatome), pancreatic site (dorsal T10 dermatome), arm (C5 dermatome), hip (L1 dermatome) and knee (L4 dermatome). Three thresholds will be measured: sensation (stimulation just felt, VAS = 1), pain detection (PDT, pricking pain just felt, VAS = 5) and pain tolerance threshold (PTT, burning pain, VAS = 7). These quantitative sensory testing (QST) techniques in relation to pancreatitis have been described in detail previously (Buscher *et al*., 2006 [28]).

### Deep muscle stimulation

The pressure pain tolerance threshold (PPTT) will be determined on the quadriceps muscle and on the same localizations as the tetanic electrical skin stimulation by pressing an electronic pressure algometer (Somedic AB, Stockholm, Sweden) on the muscle group. The probe has a surface area of 1cm^2^. The pressure will be increased at a rate of 30 kPa/sec until the PTT (VAS = 7) is reached.

The patients will be instructed to press a button when PTT is reached.

PPTT is determined to be the average of 2-3 consecutive assessments separated by two-minute intervals.

The assessment parameter will be mean value of PPTT in kPa.

## Cold pressor test in combination with pressure stimulation

In this combined test, the patient will rate the pain evoked by pressure both before and immediately after application of the cold pressor test.

The test will therefore consist of three parts (the details of the cold pressor test are outlined below:

1. PPTT determination as described above on the left quadriceps muscle.
2. Cold Pressor test on the right hand
3. Repetition of the PPTT determination on the left quadriceps muscle.

Cold pressor test: The right hand is immersed in ice-chilled water (1.0°C ± 0.3°C) that is continuously stirred by a pump. The patient will be told to remove the hand from the water after 2 minutes of immersion - or sooner if the pain is considered to be intolerable. The patients continuously rate the pain intensity during the cold pressor test with the use of an electronic visual analogue scale coupled to a computer with software designed for this purpose. From the data obtained, the peak-pain intensity (VAS_peak_), and the average area under the pain intensity-time curve (VAS_AUC_), will be determined (Jones et al., 1988 [30]). Immediately after the cold pressor test, the patients will rate the discomfort experienced during the test by use of a visual analogue scale. If the patient withdraws their hand sooner than two minutes, due to intolerable pain, the VAS will be considered to be 10 for the remaining time.

The assessment parameters will be VAS_AUC_ (for 2 min or shorter duration), VAS_peak_ (during the 2 min) (in mm) and time to reach “intolerable pain”, for example before reaching the 2 min goal (in sec).

# ADVERSE EVENT REPORTING

The methods for collecting and reporting adverse events are described below.

#### Definitions

The definitions of adverse events (AEs), adverse reactions (ARs), serious adverse events/reactions (SAEs/ARs) and suspected unexpected serious adverse reaction (SUSAR) are given below. It is of the most importance that all staff involved in the study is familiar with the content of this section. The principal investigator is responsible for ensuring this.

Adverse event and adverse reaction

An adverse event (AE) is the development of an undesirable medical condition or the deterioration of a pre-existing medical condition following or during exposure to a pharmaceutical product, whether or not considered causally related to the product. An undesirable medical condition can be:

- Symptoms (e.g., nausea, chest pain)
- Signs (e.g., tachycardia, enlarged liver)
- Abnormal test results of an investigation (e.g., laboratory findings, electrocardiogram).

In clinical studies, an AE can include an undesirable medical condition occurring at any time.

If any causality between the AE and the investigational drug is expected the AE is termed an adverse reaction (AR).

Serious adverse event and serious adverse reaction

A serious adverse event or reaction (SAE/SAR) is an adverse event or adverse reaction occurring during any study phase and at any dose of the investigational product, comparator or placebo, fulfilling one or more of the following criteria:

- Results in death
- Is immediately life-threatening
- Requires in-patient hospitalisation or prolongation of existing hospitalisation
- Results in persistent or significant disability or incapacity
- Is a congenital abnormality or birth defect

The causality of serious adverse events (i.e. their relationship to study treatment) will be assessed by the investigator(s), who in completing the relevant case report form must answer “yes” or “no” to the question: “Do you consider that there is a reasonable possibility that the event may have been caused by any of the following: – study medication? – other medication”?

**Suspected Unexpected Serious Adverse Reaction**

A suspected unexpected serious adverse reaction (SUSAR) is a serious adverse reaction with likely relationship to study treatment, which could not be expected from previous information of the investigational drug as described in the summary of the product characteristics.

#### Recording of adverse events

Information about adverse events will be collected from the first administration of any investigational product until the end of the study. Adverse events spontaneously reported by the patient and/or in response to an open question “have you had any health problems during the study since previous visit” from the study personnel or revealed by observation will be recorded at each visit from the first administration of any investigational product until the end of the study.

Any deterioration in laboratory values and vital signs need not be reported as AEs. However, abnormal values that meet criteria for an SAE or cause discontinuation of investigational product must be reported as SAE or AE and recorded in the CRF.

The time/date when the AE started and disappeared, maximum intensity, action taken with regard to investigational product, causality rating (yes or no), outcome, if AE caused patient to discontinue the study and whether or not it constitutes an SAE will be reported in the CRF for each AE.

The causality of non-serious AEs (i.e., their relationship to investigational product) will be assessed in the same way as for SAEs.

The intensity rating is defined as:

1 = mild (awareness of sign or symptom, but easily tolerated)

2 = moderate (discomfort sufficient to cause interference with normal activities)

3 = severe (incapacitating, with inability to perform normal activities)

It is important to distinguish between serious and severe AEs. Severity is a measure of intensity whereas seriousness is defined by the criteria as described above. An AE of severe intensity need not necessarily be considered serious. For example, nausea that persists for several hours may be considered severe nausea, but not a SAE.

Any AE not resolved at the last assessment or discontinuation of the study will be followed up within two weeks and thereafter as long as the investigator consider necessary.

The same parameters as for AEs / SAEs will be recorded in the case a SUSAR should occur during the study period.

#### Reporting of serious adverse events / averse reactions

Investigators and other site personnel must immediately inform sponsor of any SAE / SAR that occurs in the course of the study. The sponsor reports all SAEs / SARs to the Regulatory Authority and the Independent Ethics Committee once a year together with a security report.

Follow-up information on SAEs / SARs must also be reported by the investigator to the sponsor.

If a non-serious AE becomes serious, this and other relevant follow-up information must also be provided to sponsor as described above. All SAEs / SARs will be recorded in the CRF.

#### Reporting of suspected unexpected serious adverse reactions

In the case a SUSAR should occur during the study period the investigator and/or sponsor are responsible for informing the Regulatory Authority immediately and within 7 days for SUSARS resulting in death or causing life threatening conditions. All other SUSARs must be reported within 15 days. Appropriate follow up information must be reported within 8 days from the primary report. Reports will be reported electronically directly to the Regulatory Authority. Pfizer will be informed of eventual SUSARs as well. All SUSARs are recorded in the CRF.

- 1. **Reports at the end of the study**

At the end of the study, sponsor will report all AEs, SAEs, SARs and SUSARs in a final report for the Regulatory Authority and the Independent Ethics Committee.

# STATISTICAL METHODS AND DATA ANALYSIS

## Sample Size Determination

**9.1.1 Clinical Study**

The size of the sample is based on operating characteristics curves. The minimal difference *D* between the two treatments rejecting the null hypothesis is estimated to 25, corresponding to 25% reduction in pain. Estimated deviation is 30 (worst case scenario). Given *n* is the size of each group (placebo and pregabalin) and *a* is the number of the groups:

Φ^2^ = (*n***D*^2^) / (2**a**σ^2^ ) = (*n**25^2^) / (2*2*30^2^) = *n**0.174

Efficacy Analysis:

Calculation of Φ for different values of *n* and locating the corresponding β in the operating characteristics curves gives (α=0.05):

| N | Φ^2^ | Φ | a(n-1) | β | 1-β |
| --- | --- | --- | --- | --- | --- |
| 22 | 2,26 | 1,50 | 24 | 0.055 | 0.945 |
| 23 | 2,60 | 1,61 | 28 | 0.05 | 0.95 |
| 24 | 2,95 | 1,72 | 32 | 0.035 | 0.965 |

A test sample of 24 patients in each group (placebo and pregabalin) will provide an acceptable statistical power for the clinical arm. In total 48 patients must complete the study. Assuming a typical drop-out rate of ca. 25%, this means that some 60 patients will be recruited into the study.

**9.1.2 Experimental Study**

The experimental branch also includes 60 patients. The reliability and sensitivity of the described experimental tests are excellent (Staahl *et al*. 2006b [31]) Historically, 15-20 patients have been sufficient to reveal the effect of strong analgesics (Staahl & Drewes 2004 [32]). This was proven in a recent study where different opioids were tested in patients with chronic pancreatitis (Staahl *et al*. 2007 [33]). Based on a recent study (Buscher *et al*. 2006), typical chronic pancreatitis patients show a pain VAS of 4.4 cm (SD=2.4 cm) [34]. Based on these data, a group size of N=28 pancreatitis patients has sufficient statistical power (α=0.05, β=80%) to detect a clinically significant decrease of 2 cm in pain VAS due to treatment.

### Analysis of Primary Endpoint

All participants who have received the study medication for at least 2 weeks will be included in the analyses. Dropouts are replaced and excluded from further analyses.

All endpoints are described and presented graphically. Primary endpoints will be subjected to analysis of covariance (ANCOVA), to permit analysis of confounding variables to be included. Effect of the active treatment is evaluated and presented with 95% confidence intervals.

### Analysis of Secondary Endpoints

Changes in the secondary endpoints will be analyzed with a non-parametrical test (Mann-Whitney U) Effect of the active treatment is evaluated and presented with 95% confidence intervals.

## Safety Analysis

No formal analyses are planned for safety data. These data (adverse events, vital signs and routine laboratory tests) will be explored through the use of standard presentations of descriptive statistics.

# DATA HANDLING AND RECORD KEEPING

## Case Report Forms

As used in this protocol, the term case report form (CRF) should be understood to refer to either a paper form or an electronic data record or both, depending on the data collection method used in this trial.

A CRF is required and should be completed for each included subject. It is the investigator's responsibility to ensure completion and to review and approve all CRFs. CRFs must be signed by the investigator or by an authorized staff member. These signatures serve to attest that the information contained on the CRFs is true. At all times, the investigator has final personal responsibility for the accuracy and authenticity of all clinical and laboratory data entered on the CRFs.

## Record Retention

To enable evaluations and/or audits from regulatory authorities, the investigator agrees to keep records, including the identity of all participating subjects (sufficient information to link records, e.g., CRFs and hospital records), all original signed informed consent forms, copies of all CRFs, serious adverse event forms, source documents, and detailed records of treatment disposition. All records will be kept at least 5 years after termination of the study.

# ETHICS

## Ethical Conduct of the Trial

The trial will be performed in accordance with the protocol, International Conference on Harmonization Good Clinical Practice guidelines, and applicable local regulatory requirements and laws. The trial has been submitted for the independent ethics committee.

## Subject Information and Consent

The informed consent form must be in compliance with ICH GCP, local regulatory requirements, and legal requirements.

The investigator must ensure that each trial subject is fully informed about the nature and objectives of the trial and possible risks associated with participation. The investigator will obtain written informed consent from each subject or the subject's legally acceptable representative before any trial-specific activity is performed. The investigator will retain the original of each subject's signed consent form.

- 1. **Ethical considerations**

The patients participating will obtain no direct personal benefit, however patients receiving active study treatment (pregabalin) may experience pain relief during the investigation period. It is the hope that results conducted from the study will provide new insight to pain mechanisms and future treatment options for the chronic pancreatitis patients. By the opinion of the principal investigator these advances is superior to eventually risk or discomfort obtained during the study.

# FINANCIAL SUPPORT AND INSURANCE

# The study will be conducted as an Investigator initiated Study, with financial support from Pfizer. Patients and healthy controls participating in the study are insured in case of study related accident or death should occur as requested by law.

# PUBLICATION OF STUDY RESULTS

All publication rights belong to the principal investigator. Positive as well as negative trial results will be published in international peer-reviewed journals in the field of pain, neurophysiology and gastroenterology. A primary author will be subscribed according to the Vancouver system. A report will be submitted for the Independent Ethics Committee (CMO) at the end of the study as requested by law. The trial will be reported to Netherlands Clinical Trial Register .

REFERENCES

1. Wilder-Smith CH, Hill L, Osler W and O'Keefe S. Effect of tramadol and morphine on pain and gastrointestinal motor function in patients with chronic pancreatitis. Dig. Dis. Sci. 1999; 44:1107-16.
2. Warshaw AL, Banks PA and Fernandez-Del Castillo C. AGA technical review: treatment of pain in chronic pancreatitis. Gastroenterology 1998; 115: 765-76.
3. Dimcevski G, Schipper KP, Tage-Jensen U, Funch-Jensen P, Krarup AL, Toft E, Thorsgaard N, Arendt-Nielsen L and Drewes AM. Hypoalgesia to experimental visceral and somatic stimulation in painful chronic pancreatitis. Eur J Gastroenterol Hepatol 2006; 18: 755-64
4. Drewes AM, Reddy H, Pedersen J, Funch-Jensen P, Gregersen H and Arendt-Nielsen L. Multimodal pain stimulations in patients with grade B oesophagitis. Gut 2006; 55: 619-29.
5. Drewes AM, Schipper KP, Dimcevski G, Petersen P, Andersen OK, Gregersen H and Arendt-Nielsen L. Multimodal assessment of pain in the esophagus: a new experimental model. Am J Physiol Gastrointest Liver Physiol. 2002; 283(1):G95-103.
6. Staahl C, Dimcevski G, Andersen SD, Thorsgaard N, Arendt-Nielsen L and Drewes AM. Oxycodone shows better effect than morphine for experimental pain in patients with chronic pancreatitis. Pain 2006a; 123: 28-36.
7. Drewes AM, Pedersen J, Liu W, Arendt-Nielsen L, Gregersen H. Controlled mechanical distension of the human oesophagus: sensory and biomechanical findings. Scand J Gastroenterol. 2003a; 38(1):27-35.
8. Arendt-Nielsen L, Frøkjær JB, Staahl C, Graven-Nielsen T, Huggins JP, Smart TS and Drewes AM. The effects of gabapentin on experimental somatic pain and temporal summation. Regional Anesthesia and Pain Medicine 2007; 32(5): 382-88.
9. Baron R.: Neuropathic Pain – The Long Path from Mechanisms to Mechanism-Based Treatment. The International Journal of Pain Medicine and Palliative Care 2001; 1(1):
10. Johnson FN, Johnson RD, and Armer M.: Gabapentin in the Treatment of Neuropathic Pain. Rev. Contemp. Pharmacother., 2001; 12: 125-211.
11. Nicholson B.: Gabapentin use in neuropathic pain syndromes. Acta. Neurol. Scand. 2000: 101: 359-37
12. Ben-Menachem E. Pregabalin pharmacology and its relevance to clinical practice. Epilepsia. 2004; 45 Suppl 6:13-8.
13. Dworkin RH, Corbin AE, Young JP Jr, Sharma U, LaMoreaux L, Bockbrader H, Garofalo EA and Poole RM. Pregabalin for the treatment of postherpetic neuralgia: a randomized, placebo-controlled trial. Neurology. 2003; 60(8): 1274-83.
14. Sabatowski R, Galvez R, Cherry DA, Jacquot F, Vincent E, Maisonobe P, Versavel M; 1008-045 Study Group. Pregabalin reduces pain and improves sleep and mood disturbances in patients with post-herpetic neuralgia: results of a randomised, placebo-controlled clinical trial. Pain. 2004; 109(1-2):26-35.
15. Rosenstock J, Tuchman M, LaMoreaux L andSharma U. Pregabalin for the treatment of painful diabetic peripheral neuropathy: a double-blind, placebo-controlled trial. Pain. 2004; 110(3):628-38.
16. Bockman DE, Buchler M, Malfertheiner P and Beger HG. Analysis of nerves in chronic pancreatitis. Gastroenterology. 1988; 94(6):1459-69.
17. Di Sebastiano P, di Mola FF, Bockman DE, Friess H and Buchler MW. Chronic pancreatitis: the perspective of pain generation by neuroimmune interaction. Gut. 2003; 52(6):907-11.
18. Dimcevski G, Sami SAK, Funch-Jensen P, Le Pera D, Valeriani M, Arendt-Nielsen L and Drewes AM. Pain in Chronic Pancreatitis: The Role of Reorganization in the Central Nervous System. Gastroenterology 2007; 132: 1546-56.
19. Dimcevski G, Staahl C, Andersen SD, Thorsgaard N, Funch-Jensen P, Arendt-Nielsen L and Drewes AM. Assessment of Experimental Pain from Skin, Muscle and Esophagus in Patients with Chronic Pancreatitis. Pancreas 2007; 35(1): 22-9.
20. Piyapolrungroj N., Li C., Bockbrader H., Liu G and Fleischer D.: Mucosal Uptake of Gabapentin (neurontin) vs. Pregabalin in the Small Intestine. Pharm Res. 2001; 18(8): 1126-30.
21. Freynhagen R, Baron R, Gockel U and Tölle TR. Pain *DETECT*: a new screening questionnaire to identify neuropathic components in patients with back pain. Curr Med Res Opinions. 2006: 22(10): 1911-20.
22. Farrar JT, Young Jr JP, LaMoreaux L, Werth JL and Poole RM. Clinical importance of changes in chronic pain intensity measured on an 11-point numerical pain rating scale. Pain. 2001; 94(2): 149-158.
23. Bloechle C, Izbicki JR, Knoefel WT, Kuechler T and Broelsch CE. Quality of life in chronic pancreatitis-results after duodenum-preserving resection of the head of the pancreas. Pancreas. 1995; 11(1): 77-85.
24. Etemad B and Whitcomb DC. Chronic pancreatitis: diagnosis, classification and new genetic developments. Gastroenterology. 2001; 120(3): 682-707.
25. Randinitis EJ, Posvar EL, Alvey CW, Sedman AJ, Cook JA and Bockbrader HN. Pharmacokinetics of pregabalin in subjects with various degrees of renal function. J Clin Pharmacol. 2003; 43(3): 277-83.
26. Drewes AM, Schipper KP, Dimcevski G, Petersen P, Andersen OK, Gregersen H and Arendt-Nielsen L. Multi-modal induction and assessment of allodynia and hyperalgesia in the human oesophagus. Eur J Pain. 2003c; 7(6): 539-49.
27. Drewes AM, Gregersen H and Arendt-Nielsen L. Experimental pain in gastroenterology: A reappraisal of human studies. Scand J Gastroenterol 2003b; 38(11): 1115-30.
28. Buscher HC, Wilder-Smith OH and van Goor H. Chronic pancreatitis patients show hyperalgesia of central origin: a pilot study. Eur J Pain. 2006; 10(4): 363-70.
29. Bharucha AE. PRO: Anorectal Testing Is Useful in Fecal Incontinence. The American Journal of Gastroenterology 2006; 101(12): 2679-81.
30. Jones SF, McQuay HJ, Moore RA and Hand CW Morphine and ibuprofen compared using the cold pressor test. Pain 1988; 34(2):117-22.
31. Staahl C, Reddy H, Andersen SD, Arendt-Nielsen L and Drewes AM. Multi-modal and Tissue-differentiated experimental pain assessment. Reproducibility of a new concept for assessment of analgesics. Basic Clin Pharmacol Toxicol. 2006b; 98: 201-211.
32. Staahl C and Drewes AM. Experimental human pain models: a review of standardised methods for preclinical testing of analgesics. Basic Clin Pharmacol Toxicol. 2004; 95(3): 97-111.
33. Staahl C, Dimcevski G, Andersen SD, Thorsgaard N, Christrup LL, Arendt-Nielsen L and Drewes AM. Differential effect of opioids in patients with chronic pancreatitis: an experimental pain study. Scand J Gastroenterol. 2007; 42(3): 383-90.
34. Buscher H, Wilder-Smith OH, van Goor H. Chronic pancreatitis patients show hyperalgesia of central origin: A pilot study. Eur J Pain. 2006; 10(4): 363-70
